# Hybrid Filaments from *Saccaharina lattisima* Biomass: Engineering of Alginate Properties with Maleic Anhydride Grafted Linseed Oil

**DOI:** 10.3390/polym13050836

**Published:** 2021-03-09

**Authors:** Martin Sterner, Ulrica Edlund

**Affiliations:** Fibre and Polymer Technology, KTH Royal Institute of Technology, Teknikringen 56, SE-100 44 Stockholm, Sweden; msterne@kth.se

**Keywords:** filament, fiber, alginate, linseed oil, maleic anhydride, macroalgae, *Saccharina*

## Abstract

Linseed oil was graft modified with maleic anhydride and introduced into alginate by co-extrusion, producing alginate hybrid filaments. A straightforward grafting of maleic anhydride onto the oil backbone produced the modified oil. Additional esterification with *n*-dodecanol was also investigated. The structures of the modified oils were verified with 2D-NMR. The modified oil was mixed with alginate and extruded into CaCl_2_, forming thin filaments with diameters in the 130–260 μm range. The impact of oil integration into the alginate filaments was assessed, with special emphasis on stress-at-break, and compared to values predicted by an empirical model relating the “stress to alginate concentration” ratio to prevailing conditions during filament drawing. Analogous alginate filaments were prepared with hydrochloric-, oxalic- and phytic acid calcium salts for comparison with alginate–oil hybrids to reveal the induced impact, with respect to the composition and charge, on the tensile performance.

## 1. Introduction

A transition from a fossil-dependent society to a circular economy requires new solutions that rely on bio-based resources. Alginate is a bio-based and highly charged polyelectrolyte present in the cell walls of brown algae. Alginate normally constitutes 20−40% of the brown algal dry weight. Brown macroalgae (*Saccharina latissima*) grow naturally and are also cultivated at high yield along the Swedish west coast [1,2,3] and hence present a promising biomass for alginate recovery via biorefining [4]. Alginate is composed of two uronic acid structural units: mannuronic (M) and guluronic (G) acid. The ability to crosslink in the presence of certain metal ions makes alginate an excellent candidate for forming gels which are strong even at low concentration [5,6,7]. Strong and flexible gels prepared from crosslinking alginate with calcium ions can also be drawn to induce chain orientation and dried into strong alginate filaments [8]. Stiff alginate filaments have tensile stress-at-break properties in the same order of magnitude as cotton cellulose or nylon [8,9,10,11,12]. The strength of the filaments come from the fact that alginate are long polyelectrolytes with high charge density that bind specifically to calcium. On the contrary, small organic molecules with similar or higher charge density compared to alginate generally do not give any significant tensile strength to the material it forms when binding calcium. Both alginate polyelectrolytes and smaller anionic charged molecules however show affinity to calcium ions and can be held together by their common affinity for calcium ions and then form hybrid materials with altered properties. For instance, charged molecules with a more hydrophobic backbone or ions with higher charge density can be introduced and held in place by the affinity to calcium ions. An effective method to make hybrid filaments of alginate and other components is to coextrude them into calcium chloride solution. When a new component is added it is important to know how that component will alter the tensile performance of the filaments. In this study, different maleic anhydride grafted linseed oils were used as model compounds representing small charged molecules with different hydrophobicity and charge densities. To produce more hydrophobic model compounds, dodecanol esters of the maleic anhydride grafted linseed oils were also produced. As well the high charge density salts hydrochloric-, oxalic- and phytic acid calcium salt complement the grafted linseed oils as mixing components to alginate, the two later representing anions with very high calcium affinity, while the chloride ion has a low calcium affinity. The knowledge of the effect by introduction of charged and hydrophobic hybrid components can be of use for several applications. For alginate used alone or as a composite for tissue engineering the hydrophilicity of the alginate promote tissue interaction [13,14,15,16]. On the contrary, the inherent hydrophilicity of alginate, presents a challenge when alginate filaments are used to reinforce bulk plastics, of which a vast majority are hydrophobic. The difference in hydrophilicity impairs the compatibility at the interface of the constituents and thus the mechanical performance [17,18].

Our aim was to engineer the properties of alginate by decoration with hydrophobic elements as to enhance the tensile performance and shed light on the coextrusion effects and composition–property relationships. Hybrid filaments prepared from alginate recovered from *Saccharina lattissima* biomass in a biorefining process were compared to the corresponding expected tensile strength of pure alginate to evaluate the effect of immobilized components in drawn alginate filaments (drawing being a necessity for high stiffness and tensile strength). The expected pure alginate tensile strength was calculated with a previously derived empirical model based on the alginate concentration in the filaments and drawing stress during drying.

## 2. Materials and Methods

### 2.1. Materials

Sodium alginate was extracted from the brown algae *Saccharina latissima* using a cyclic fractionation process in which diluted sodium citrate was used in 18 sequential extractions from the same biomass [4]. The alginate fraction used in this work was recovered, by acid precipitation followed by centrifugation, from the 16th extraction cycle. The algae were cultivated on the Swedish west coast and harvested on 17 May 2016, from a cultivation site at the Sven Lovén Centre for Marine Science (University of Gothenburg). The precise extraction protocol is described in a previous work [8]. Commercial sodium alginate, sodium hydroxide (≥97% ACS reagent, CAS: 9005-38-3), maleic anhydride (MA) (≥99.0% puriss), *n*-dodecanol (DD) (≥98%, reagent grade), calcium chloride dihydrate (CaCl_2_) (≥99% ACS reagent), sodium oxalate (≥99.5% ACS reagent), and phytic acid (≥90% phosphorus basis) were purchased from Sigma-Aldrich, Sweden. Deuterated chloroform (CDCl_3_) (99.96%) was purchased from Cambridge Isotope Laboratories, U.S.A. Linseed oil was purchased from Slöjdetaljer, Sweden, and produced by Eskil Åkerberg AB, Staffanstorp, Sweden.

### 2.2. Grafting of Linseed Oil

**Scheme 1:**
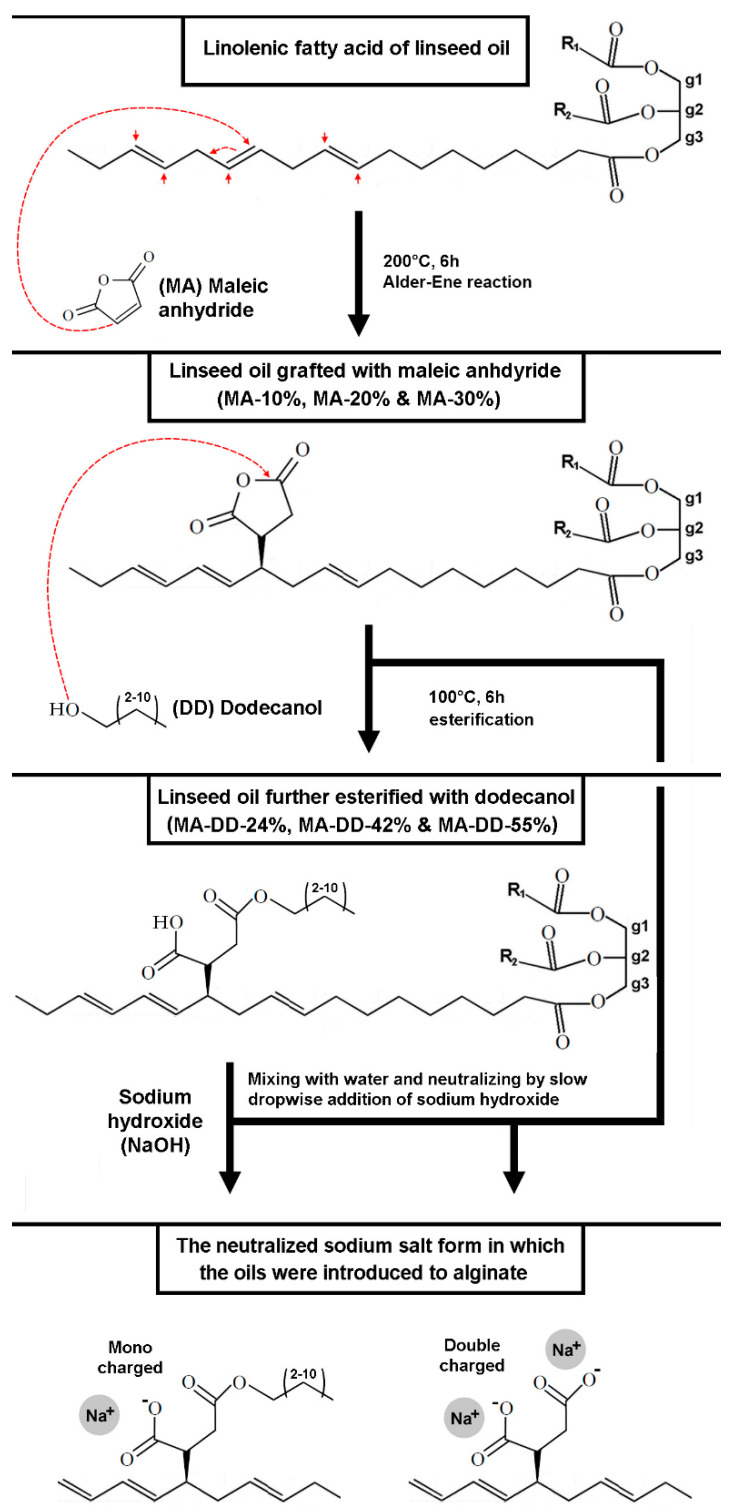
Schematic illustration of the preparation of grafted linseed oil.

Linseed oil was grafted with maleic anhydride (MA) to immobilize succinic anhydride (SA) groups onto the oil backbone (Scheme 1). The grafting reaction was carried out at 200 °C for 6 h in closed round bottle flasks with rigorous stirring. The flasks were filled to approximately 80% of their volume with the initial air present during grafting reaction. The grafting proceeds mainly by the Alder–Ene reaction [19]. In some cases, the grafted oil was further reacted with *n*-dodecanol (DD) to form succinic half esters. DD was added at a molar ratio of 1:1 with respect to the amount of added MA and kept at 100 °C for 6 h in glass vials with extensive stirring. The reaction mixture was neutralized by slow dropwise addition of sodium hydroxide and diluted to a concentration of 6% (*w*/*w*) in deionized water. Upon the addition of water, the grafted anhydride groups hydrolyzed and formed succinic acid groups with two carboxylic acids each. The succinic half esters, on the other hand, only have one charged group each. The proportions of the components are given in Table 1 along with the calculated charge density of each oil. The grafted oils are denoted as MA-X% where X represents the weight percent of added MA. The oils further reacted with DD are denoted as MA-DD-Y% where Y represents the combined weight percent of MA and DD. The charge densities (Table 1) were calculated assuming that all acid groups are dissociated. In comparison, alginate has a charge density of 4.7 mol charges/kg.

### 2.3. Filaments of Alginate, Salts, and Grafted Linseed Oil

Filaments were prepared from two grades of alginate in parallel: Commercial alginate from Sigma-Aldrich (Sweden) and alginate extracted in an in-house developed cyclic biorefinery fractionation process [4] (herein denoted as extracted alginate). Alginate solutions, 2% or 4% (*w*/*w*), were mixed with grafted linseed oil (prepared as described in Section 2.2) in a 2:1 ratio with respect to the weight of alginate and the weight of oil. The effective concentration in the mixed solutions was 3% (*w*/*w*) commercial alginate with 1.5% (*w*/*w*) oil, and 1.71% (*w*/*w*) extracted alginate with 0.86% (*w*/*w*) oil, respectively. In parallel, reference filaments were prepared from alginate mixed with salts: 2% (*w*/*w*) alginate with 0.13% (*w*/*w*) di-sodium oxalate (0.010 M) or 0.21% (*w*/*w*) octa-sodium phytate (0.0025 M). Reference solutions, without oil, of commercial alginate (3 and 5%) and extracted alginate (2%) were also prepared.

**Scheme 2:**
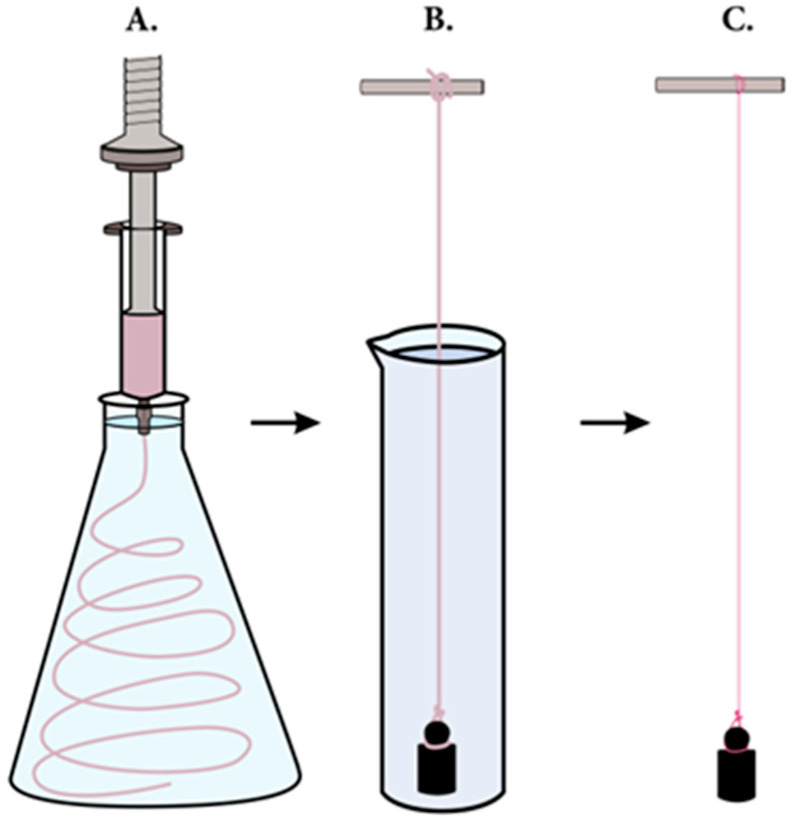
Schematic illustration of the preparation of alginate filaments: (**A**) extrusion, (**B**) filament drawing, and (**C**) filament drying.

The solutions were loaded into plastic syringes and extruded through a metal-nozzle (end diameter of 1 mm) connected to the syringe. The extrusion speed was controlled with a ProSense NE300 (Germany) screw syringe pump (Scheme 2, step A). The nozzle-end was placed approximately 1 cm below the liquid surface into a 20 cm high conical broad necked flask filled with crosslinking solutions (CaCl_2_, 0.25 M or 0.09 M). The extrusion speed was set to 3 mL/min for samples with commercial alginate and 0.4 mL/min for samples with extracted alginate. The generated gel filaments were left in the crosslinking solution for 2.5 h and then individually mounted to plummets and drawn in measurement cylinders filled with 0.02 M CaCl_2_ (Scheme 2, step B). The plummets had an apparent weight of 30 g in water (34.4 g in air). The filaments were removed from the cylinders after 2.5 h and dried overnight still with the plummets attached (Scheme 2, step C). The dry alginate filaments were cut into 4 cm long pieces and further dried in a desiccator for 3 days before weighing.

Grafted oil and phytic or oxalic anions all form insoluble calcium salts and will therefore precipitate if they are co-extruded with alginate into a CaCl_2_ solution. In other cases when filaments were generated, a high concentration of CaCl_2_ (0.09 M or 0.25 M) was used during the whole course of the filament generation, which made CaCl_2_ salt enter the filaments. The final CaCl_2_ salt concentrations in the filaments were 7% (*w*/*w*) and 30% (*w*/*w*) when generated in CaCl_2_ solutions with a concentration of 0.09 M and 0.25 M, respectively. The integrated amount of CaCl_2_ salt was determined by comparing the weight of an alginate sample which was previously immersed in CaCl_2_ solution (0.25 M or 0.09 M) with an alginate sample previously immersed in a CaCl_2_ solution of lower concentration (0.02 M) where a great deal fewer excess ions entered the gel.

### 2.4. Tensile Testing

Filament specimens with a length of 4 cm (prepared as described in Section 2.3) were conditioned at 50% relative humidity for 3 days before tensile testing. Tensile testing was performed with an Instron 5566 instrument and Bluehill software was used for the test control and collection of data. The filaments were fixed by gluing 1 cm of each end into a glass pipe filled with epoxy glue and then clamping the pipes into the grips of the instrument. A 500-N load cell was used for the testing.

### 2.5. Nuclear Magnetic Resonance, NMR

^1^H-NMR with deuterium oxide as the solvent was used for the characterization of alginate. The grafted linseed oil was also analyzed by NMR, ^1^H-NMR, and ^13^C-NMR with deuterated chloroform as the solvent. To assign all peaks of the grafted oil, the ordinary NMR was also complemented by Heteronuclear Multiple Bond Correlation (HMBC) and Heteronuclear single quantum coherence (HSQC) analysis. Alginate required a mild degradation to give good spectral resolutions. The mild degradation was done by dissolving alginate in water and adjusting the pH to 3 by the addition of either dilute sodium hydroxide or hydrochloric acid. Samples were then degraded at 100 °C for 1 h, cooled and then neutralized to pH 7 with sodium hydroxide. The samples were left to dry under a gentle stream of air at room temperature for approximately one day. Samples of approximately 2% dried material were dissolved in deuterium oxide and transferred to NMR tubes. In all cases, the NMR tubes had 5-mm diameters and the spectra were recorded at 500 MHz on a Bruker DMX-500 NMR spectrometer. MestReNova software was used for data acquisition.

### 2.6. Rheology Measurements

Sodium alginate 1% (*w*/*v*) was dissolved in deionized water and frequency sweeps were run in a TA Instruments Discovery HR-2 rheometer. A 25 mm diameter steel plate was used, with a 100 µm plate gap and an applied pressure of 4 Pa. The storage and loss moduli were measured between 10–1.2 and 101.5 Hz. Trios v.4.21 software was used for data acquisition and calculations.

### 2.7. pH Measurements

A VWR SympHony SB70P pH-meter equipped with a Hamilton Biotrode electrode was used to measure the pH of the emulsions of 6% grafted linseed oil during the process of hydrolyzing them with sodium hydroxide. CertiPur^®^ disodium hydrogen phosphate/potassium dihydrogen phosphate (pH 7.0) and CertiPur^®^ potassium hydrogen phthalate (pH 4.01) solutions from Merck were used as the pH calibration standards.

## 3. Results and Discussion

Alginate–linseed oil hybrid filaments were prepared. We used simple one-pot chemistry to graft functionalize linseed oil and thereby make the oil water miscible and compatible with alginate. Linseed oil was chosen as the bio-based feedstock since it is very accessible [20] in Sweden and contains many fatty acids which provide hydrophobicity and enable easy grafting chemistry. MA was chosen as the grafting linker as it binds to alkenes at moderate temperatures [21]. The grafting preference for alkenes was a reason to choose linseed oil over other oils, since linseed contains many unsaturated fatty acids, which are rich in ene-groups. It is possible to generate MA catalytically from the highly accessible bio-based molecule furfural [22] which is another reason for choosing MA as the monomer. Some alginate samples were further reacted with DD, which was also chosen based on the potential to generate the molecule from a bio-based feedstock (lauric acid) [22,23,24].

A challenge when working with inherently heterogeneous biomacromolecules such as alginate and linseed oil is characterizing the structural change during the reaction. We chose to face this challenge with different NMR techniques and to utilize model studies to reveal the structures of pristine and modified material components.

### 3.1. Characterization of Alginate

**Figure 1:**
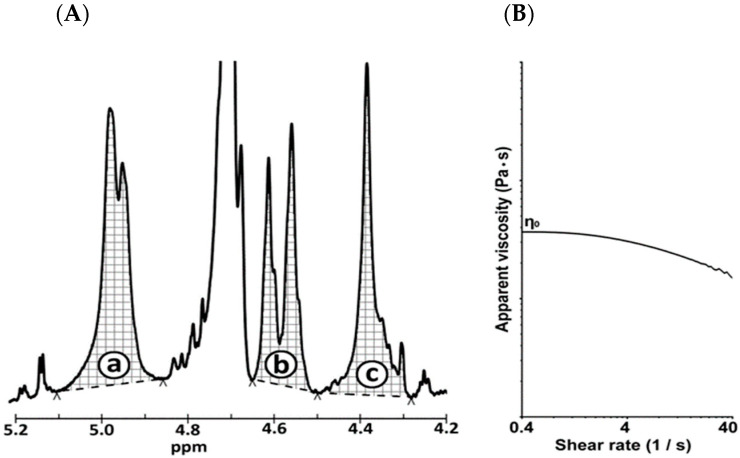
(**A**) Areas of peaks a, b and c in the ^1^H-NMR spectrum of extracted alginate used to calculate the uronic acid composition with Equations (1) and (2). (**B**) A typical viscosity curve of a rheological frequency sweep, from which the zero-shear viscosity is determined. The alginate molecular weight (M_w_) is approximated from η_0_ using the relationships from Rinaudo and Graebling [25].

Two different alginates were used: a commercial grade and an alginate fraction recovered in a cyclic fractionation biorefining process of the brown algae *Saccharina latissima*. The molecular weight (M_w_) and uronic acid composition of both grades were determined. The uronic acid composition of alginate was determined by NMR, using the peak areas of a, b, and c (Figure 1). The share of guluronic acid (G) and guluronic acid dyads (GG) was calculated from Equations (1) and (2). Commercial alginate had a G share of 58% with 36% GG dyads while extracted alginate had a G share of 79% with 55% GG dyads.

The molecular weight (M_w_) of the two used alginates was approximated by determining the zero-shear viscosity (η_0_) with rheometer frequency sweeps and utilizing relationships determined by Rinaudo and Graebling (Equations (3) and (4)) to convert η_0_ values to M_w_ [25]. In Equation (3), C[η] is the overlap concentration, [η] is the intrinsic viscosity, and η_sp_ is the specific viscosity. The parameters 0.37 and 0.1 in Equation (3) are chosen for a scenario with 0.1 M added salt [25]. We utilize a viscosity value of 0.8993 × 10^−3^ (0.1 M NaCl solution at 25 °C) to calculate η_sp_ from η_0_ [26]. Solutions with 1% (*w*/*w*) alginate were used. The complex viscosity as a function of angular frequency (rad/s) was directly determined from the frequency sweeps. The empirical Cox-Merz rule can be applied if the complex viscosity is presented as a function of angular frequency (rad/s) [27]. When applicable, the rule stipulates that the complex viscosity as a function of (rad/s) is approximately equal to the apparent viscosity (η) as a function of (1/s). The zero-shear viscosity (η_0_) can then be calculated from the apparent viscosity at the lowest shear rate of our measurements (0.4 s^−1^) where the curves have leveled out the most. For commercial alginate, η_0_ was determined as 0.015 Pa·s and the approximate Mw was 240 kDa. For extracted alginate, η_0_ was determined as 6.1 Pa·s and the approximate Mw was 2500 kDa.

(1) $$\mathrm{Share}\;\mathrm{of}\;G=\;\frac{a}{b+c}$$

(2) $$\mathrm{Share}\;\mathrm{of}\;\mathrm{GG}=\frac{c}{b+c}$$

(3) $$\eta_{sp}=C\left[\eta \right]+0.37\cdot {\left(C\left[\eta \right]\right)}^{2}+0.1\cdot {\left(C\left[\eta \right]\right)}^{3.2}$$

(4) $$\left[\eta \right]=\;2\cdot 10^{-3}\cdot \;M_{w}{}^{0.97}$$

### 3.2. Characterization of Grafted Linseed Oil

**Scheme 3:**
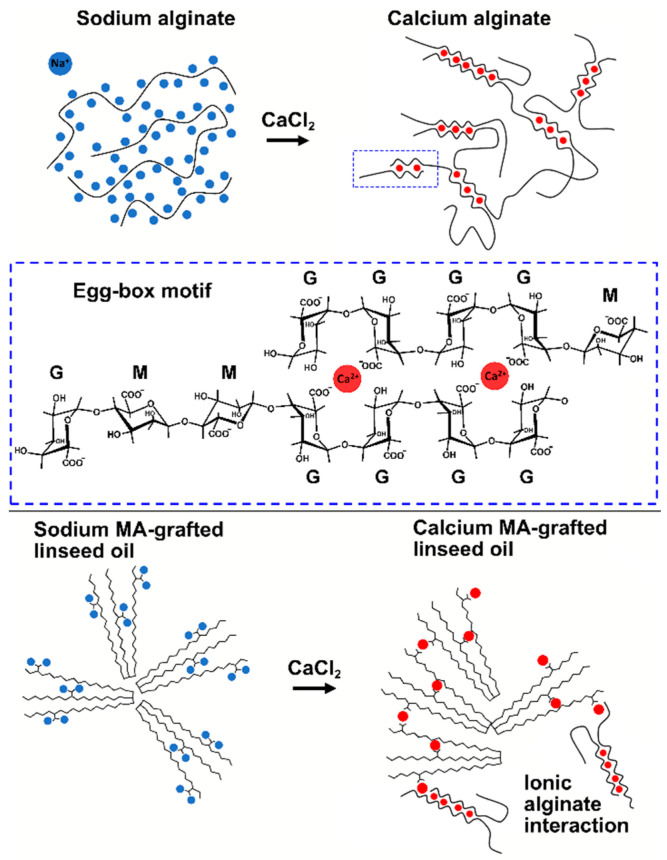
A schematic illustration of the reaction occurring during crosslinking of alginate.

The progression and outcome of the linseed oil grafting reactions (Scheme 1) were analyzed with ^1^H and ^13^C NMR. HMBC and HSQC 2D NMR were applied on three samples: pure linseed oil, the reaction products of MA-20%, and MA-DD-42% to verify the product structures. The schematic reaction occurring during subsequent crosslinking in the presence of alginate are further illustrated in Scheme 3.

**Figure 2:**
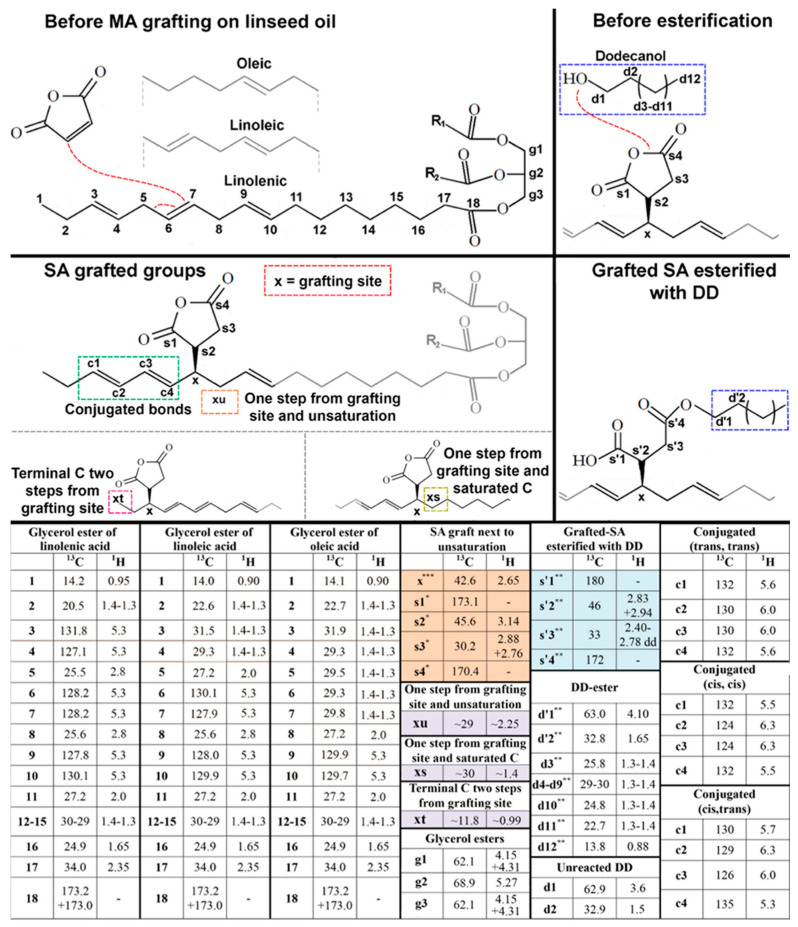
Possible structures of maleic anhydride (MA) linked to linolenic, linoleic and oleic acid. Orange color in the table denotes peak assignment data gathered from reference 28, white from ref. 29–32, blue from ref. 33 and purple is estimated from the 2D-NMR data in this study (Figure 4). * Atom belonging to SA groups. ** Atoms belonging to the succinic half ester. *** Atom at the branch point of which a SA group is attached.

The regions of the ^1^H and ^13^C NMR-spectra in which the peaks indicate a successful grafting of MA, resulting in covalently immobilized SA groups onto the oil backbone, were found based on a study of ^13^C-succinic anhydride bound to model alkenes by ^13^C NMR and ^1^H NMR [28]. The ^1^H NMR and ^13^C NMR peak assignments for lipids and succinic monoesters were also found in the literature [29,30,31,32,33]. The combined information from these studies provided the basis for the peak assignments of the various analogues of grafted linseed oil listed in Figure 2, considering that several sites for maleic anhydride binding are plausible. Since the reaction proceeded at a temperature of 200 °C, it was expected that the majority of the reactions were of the Alder–Ene type and those are the scenarios described in Figure 2 [21]. It has also been suggested that the reaction of maleic anhydride is not a pure Alder–Ene reaction but also to some extent proceeds as a radical reaction [34,35].

The most apparent peak shift regions are those in direct proximity to the groups s1–s4. The significant peaks related to the reaction product of linseed grafted with DD are s’1-s’4 and the introduced peaks of the DD ester are d1-s12. The radical reactions mediating the grafting cause double bonds to shift position and generate conjugated double bond systems giving rise to peaks denoted c1-c4. All ^13^C spectra are normalized to the carbonyl peak of the glycerol ester at 173.2 ppm and all ^1^H spectra are normalized to the glycerol ester peak at 4.33 ppm.

**Figure 3:**
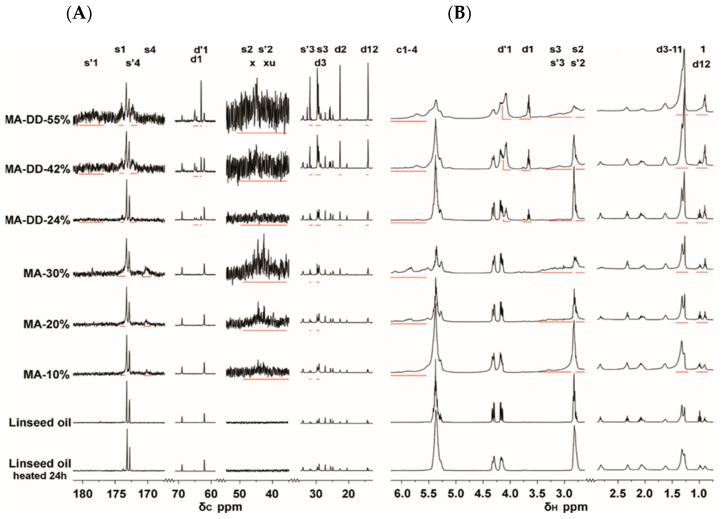
NMR spectra of (from bottom to top): linseed oil heated for 24 h at 200 °C, fresh linseed oil, linseed oil reacted with 10%, 20% and 30% maleic anhydride (MA) as described in Section 2.2, and the reaction products of the continued reaction with *n*-dodecanol (DD) as described in Section 2.2. (**A**) ^13^C NMR with all spectra normalized to the carbonyl peak of the glycerol ester at 173.2 ppm. (**B**) ^1^H spectra normalized to the glycerol ester peak at 4.33 ppm. The important shift regions pointed out in Figure 4, in proximity to the grafted groups, are shown. The shift segments have different vertical scales to allow visual clarity of important peaks.

The peak regions of s1-s4 increase significantly with increasing amount of reacted MA (Figure 3). This is consistent with the peak regions of the proposed structure of MA-grafted oil shown in Figure 2. When the grafted groups are further esterified with DD, the peak regions s’1-s’4 increase and s1-s4 decrease. The most apparent decrease, given that this peak is well resolved, is that s4 disappears after the reaction with DD. The peaks d1 and d2 are assigned to esterified DD groups. The presence of peaks d’1 and d’2 indicates that not all DD has reacted. Approximately 85% of the added DD has reacted according to a comparison between the integrals of peaks d’1 and d1.

**Figure 4:**
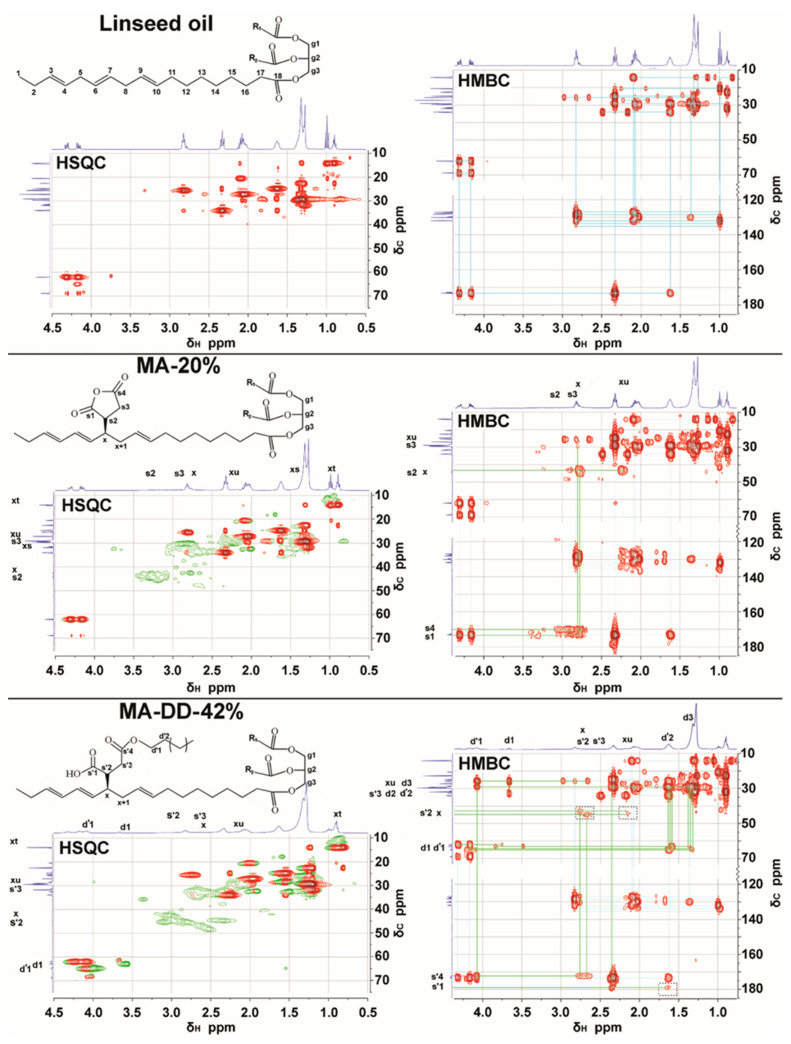
HSQC and HMBC spectra of raw linseed oil, linseed oil grafted with MA, and linseed oil grafted with MA esterified with DD. The unique regions of HSQC correlation compared to raw linseed oil are shown in green for the reacted products. For HMBC, the new correlations that are not found in the HMBC-spectrum of raw linseed oil are marked with green lines. The three frames in the HMBC spectrum of linseed oil DD esters represent amplified areas to make the peaks visible.

HMBC and HSQC NMR were used to reveal if the assumed ^1^H and ^13^C specific peaks of SA groups and DD succinic esters correlate to each other (Figure 4).

The HSQC spectrum of MA-20% oil reveals that the peak regions of s1-s4 and x correlate well with the ^13^C and ^1^H values similar to the expected structures in Figure 2 and the position of the peak areas that increased with higher amounts of added MA in Figure 3. The peaks of the carbon groups xu (graft position next to unsaturation), xs (graft position next to saturated C) and xt (graft position two steps from terminal C) in the proximity of the grafting site were determined by their positions in the HSQC spectrum. The connection between x and xu could also be seen in the HMBC spectrum. It is expected that xu would show a clear peak as it is the most common carbon that is adjacent to the graft point. If linolenic acid (which is the most common in linseed oil) is grafted by MA, four out of six combinations give rise to xu. The many small peaks and overlaid peaks are due to the many plausible structural analogues produced in the grafting, including both threo and erythro configurations of the graft groups. The HSQC spectrum of linseed oil reacted with MA and esterified with DD also shows reasonable ^13^C and ^1^H correlations between the s’1-s’4 and x peak regions. The introduced DD esters and unreacted DD also give rise to the distinct new peaks d’1 and d1. For HMBC of MA-20%, the long range ^1^H-^13^C connections are seen for s1-s4 and x regions within two or three bonds from each other. For MA-DD-20%, these connections were weaker but still visible for s’1, s’2 and s’4 and x. The correlations of s’3 to its neighbors were not seen, most likely since they were too weak. Correlations between the carboxyl groups of carboxylic acid s’4 to s’2 were observed and expected; however, a weak correlation between a carboxylic acid and s2 was observed also in the case of non-DD-esterified oil. This could be explained by the presence of small amounts of water in the reaction with linseed oil and MA, possibly causing hydrolysis of a fraction of the anhydride groups, as reported in a previous study on MA [19].

### 3.3. Tensile Properties of Alginate with Modified Linseed Oil and Calcium Salts

**Figure 5:**
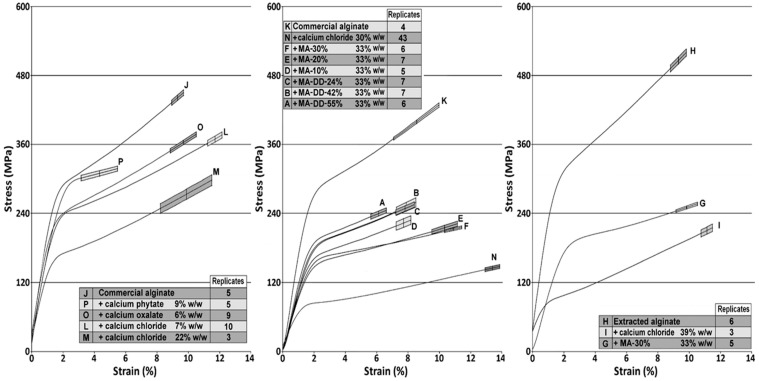
(Graphs) The average stress–strain curves of calcium alginate gel filaments prepared from alginate solutions and a blend with a salt or grafted linseed oil. The center of each error zone represents the average stress-at-break at correlated strain values. The vertical boundaries of the error zones are given by the double-sided T-distribution (90% confidence level) of the stress values. The lateral boundaries are in turn given by the corresponding strain for the upper and lower bound stress values at the point of average stress-at-break.

The addition of polyvalent cations generates materials with different mechanical properties when generating dry filaments from alginate [8]. We have previously shown that the stiffness and strain-at-break values of alginate filaments were typically higher when Al^3+^ ions were used as crosslinkers, while filaments crosslinked with Ca^2+^ had higher strain-at-break and slightly higher stress-at-break [8]. Here, we generated filaments in Ca^2+^ and introduced grafted linseed oil to characterize the effect on the tensile properties. The tensile properties of alginate mixed with grafted oils and salts are presented in Figure 5 and Table 2.

Clearly, the added grafted oils markedly weakened the filaments compared to the reference alginates without oil (Figure 5). The grafted oils are incorporated in the alginate as charge complexes with calcium salts, which makes a comparison to other calcium salts relevant.

When filaments with the incorporated calcium salts of the grafted oils are compared to filaments with other incorporated calcium salts (CaCl_2_, calcium oxalate, and calcium phytate), it appears to be the other salts which weaken alginate more. Calcium oxalate and calcium phytate are added in small amounts but still decrease the stress-at-break significantly, which reveals that the incorporation of these salts is very impactful in weakening the alginate filaments. It is interesting to compare MA-DD-55% to MA-10% (Figure 5K,J) since they have similar charge densities but the graft succinic groups are double charged in the case of MA-10%, while for MA-DD-55% the half esterified succinic groups are mono charged. The only completely soluble oil was MA-30%; in this case, the oil and alginate formed transparent solutions, while in the other cases, the blends were stable opaque emulsions. It is interesting to see if the tensile behavior of a material made with the completely soluble oil (Figure 5H) differed markedly from the other oil. The trends in the shape of the tensile test curves following the addition of calcium salts can be seen in Figure 5, but to truly address the effect of incorporated grafted oils and calcium salts, the difference in filament diameters and amount of incorporated material must also be considered. The addition of oils made it harder to control the filament thickness, so to compare the oils, it was necessary to derive a method that accounts for this difference.

We have previously developed a mathematical model that calculates the expected stress-at-break based on the drawing stress during alginate filament preparation [8]. The equation (Equation (5)) expresses stress as MPa and concentration as % (*w*/*w*). Another way to express the relationship is by instead relating the draw stress to the dry filament drawing stress (Equation (6)). The same parameters can be derived if the gel density, dry material density, and the engineering strain during drawing are known.

(5) $$\mathrm{Stress}-\mathrm{at}-\mathrm{break}=189+405\cdot \log_{10}\left(\frac{\mathrm{wet}\;\mathrm{gel}\;\mathrm{drawing}\;\mathrm{stress}}{\mathrm{alginate}\;\mathrm{concentration}\;\mathrm{in}\;\mathrm{wet}\;\mathrm{gel}}\right)\;$$

(6) $$\mathrm{Stress}-\mathrm{at}-\mathrm{break}=189+405\cdot \log_{10}\left(\frac{\mathrm{dry}\;\mathrm{filament}\;\mathrm{drawing}\;\mathrm{stress}}{\mathrm{strain}\;\mathrm{during}\;\mathrm{drawing}}\;\cdot \;\frac{\mathrm{dry}\;\mathrm{density}}{\mathrm{gel}\;\mathrm{density}}\right)\;$$

The incorporation of excess salt in the filament possibly changes the tensile properties. To reveal this effect, we calculated the potential stress-at-break for the same amount of alginate in a filament without the salt using Equation (7). Excess salt is expressed as c_salt_ (in % (*w*/*w*)).

(7) $$\mathrm{Stress}-\mathrm{at}-\mathrm{break}=189+405\cdot \log_{10}\left(\frac{\mathrm{dry}\;\mathrm{filament}\;\mathrm{drawing}\;\mathrm{stress}}{\mathrm{strain}\;\mathrm{during}\;\mathrm{drawing}}\;\cdot \;\frac{\mathrm{dry}\;\mathrm{density}}{\mathrm{gel}\;\mathrm{density}}\cdot \;\frac{1}{1-c_{\mathrm{salt}}}\;\right)\;$$

Measurements of the engineering strain during drawing were performed on different filaments from both commercial alginate and extracted alginate. Commercial alginate elongates to approximately 150%, 170% and 185% in filaments made with initial alginate concentrations of 5% (*w*/*w*), 3% (*w*/*w*), and 2% (*w*/*w*), respectively. In the case of filaments made from 2% (*w*/*w*) extracted alginate, the elongation was 162% or 170% for the two distinct filament thicknesses that resulted from using 0.02 M or 0.25 M CaCl_2_ in the crosslinking. The density of dry alginate filaments was measured to 1890 kg/m^3^. The density was measured by immersing a weighed amount of alginate filaments in a thin glass tube, filling the tube to a set height with isopropanol, and weighing it. The tube was previously weighed when filled with isopropanol to the same height (isopropanol having a known density of 786 kg/m^3^). The alginate density was calculated by comparing the weight of the tube with and without the added filaments. The alginate density of the non-dried gel filaments was approximated as 1000 kg/m^3^. A more accurate density would be to start with the density for a 0.02 M CaCl_2_ solution, i.e., 999 kg/m^3^, at room temperature and to add the density effect of the alginate concentration. When extrapolating the result by a density study on alginate, Klemenz et al. [36] observed that a 1% (*w*/*v*) increase in alginate content in a calcium alginate gel added 3 kg/m^3^ to the density (in the gel concentration range of 1–2% (*w*/*v*)). This however makes a small difference compared to the approximate value of 1000 kg/m^3^.

With Equation (7), we can compare the actual measured stress-at-break performance of the filaments with added excess salt to the expected value of alginate filaments without the excess salt. To make a better comparison, it is however better to use a similar approach on the measured stress-at-break value and adjust it to the amount of stress that the alginate would experience without the added excess calcium salt, as shown in Equation (8):

(8) $$\mathrm{Adjusted}\;\mathrm{stress}-\mathrm{at}-\mathrm{break}=\mathrm{measured}\;\mathrm{stress}-\mathrm{at}-\mathrm{break}\cdot \frac{1}{1-c_{salt}}\;\;$$

**Figure 6:**
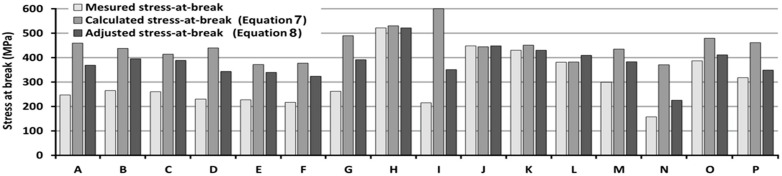
(bar diagram) The measured higher average stress-at-break (average of upper 50% of stress-at-break values). Calculated higher average stress-at-break for filaments without their incorporated excess salts (Equation (7)). Adjusted higher average stress-at-break data, with stresses calculated from the cross-section area that filaments would have without the incorporated excess salt (Equation (8)). n.m. = not measured, n.s. = no excess salt.

The drawing stress, calculated stress-at-break, and measured stress-at-break are listed in Figure 6. Since the empirical Equation (5) is derived from a dataset from which only the upper 50% of the tensile test data was used, the same upper 50% of the stress-at-break data was used for the filaments with MA-graft oil and calcium salts.

The introduction of the grafted oil to the filaments weakened alginate in all cases; however, the effect is far less dramatic than when comparable amounts of calcium salts are introduced (Figure 6 and Table 3). The values of (Δ stress-at-break by the added excess salt (%/%)) reveal that the addition has a far lower impact on the stress-at-break values compared to calcium salts, especially for MA-DD-oils (Table 3). Among the calcium salts, oxalic acid and phytic acid weaken alginate the most. For CaCl_2_, an accelerated decrease in stress-at-break seems to occur once it is incorporated at higher concentrations. The effect per added percent of salt seemingly increased when more salt was added. The sample with only 7% CaCl_2_ gives a negative Δstress-at-break, which could indicate that it contributes a little to the stress-at-break of alginate filaments. Since there is also some variation in the test and limits to how good the tensile behavior can be modeled with Equation (7), a weak trend such as this one can also be due to measurement deviations. Another property that seemingly could cause much weakening is an increased amount of absorbed moisture. The amount of absorbed moisture was tested for most of the filaments (Table 3). The moisture absorption was measured by weighing the filaments after drying in a desiccator and comparing it to their weight after three days of saturation in 50% air humidity. There was no dramatic difference in the moisture content between the samples, but the filaments to which oil had been introduced absorbed possibly less moisture. Filaments made from oils modified with DD esters and with low amount of grafting absorbed less moisture, and filament with phytic acid salt absorbed the most moisture.

In summary, grafted linseed oil was successfully formed and incorporated easily into alginate filaments. The change in tensile strength was evaluated for the alginate–oil hybrid filaments, as well as for alginate with calcium salts. Linseed oil is a bio-based co-component which may add the hydrophobization needed improve the interphase adhesion between alginate-based filaments and hydrophobic matrices, for instance in future development of bioplastics, in biocomposites and blends.

## 4. Conclusions

Renewable composite filaments were prepared from alginate, recovered in a biorefining process, and modified with linseed oil or calcium salts. The linseed oil was graft-modified with maleic anhydride to create ionic binding sites allowing for charge interaction with alginate and crosslinking upon extrusion into Ca^2+^ solution. Some samples of grafted oils were further reacted into succinic dodecanol half esters. All grafted oils formed stable blends with alginate, but only the oil with the highest graft and charge density (30% maleic anhydride) became fully water-soluble and formed transparent solutions. The introduction of grafted oils into alginate filaments decreased the stress-at-break and changed the tensile behavior of alginate. An empirical model that predicts the stress-at-break from the stress applied when drawing filaments in the preparation step was used to evaluate the effect of the added components. The predicted results were compared to the measured stress-at-break values (with the volume contribution from the excess salt excluded in both cases) to reveal the impact of each component on the mechanical performance of the filaments.

Oils with higher graft densities of succinic groups, and in some cases additional dodecanol succinic half esters, had the least impact on the tensile properties (0.2–0.4% decrease in stress-at-break per added (*w*/*w*) of oil). Alginate filaments with grafted oil hence sustain similar forces as filaments made from alginate only, but since oil is incorporated, these filaments are slightly thicker. For comparison, the introduction of CaCl_2_, calcium oxalate or calcium phytate to alginate filaments caused filament weakening with 1.2%, 2.4% and 2.6% decreases in stress-at-break per added % (*w*/*w*) of salt, respectively.

## Figures and Tables

**Table 1:** Proportions of the components of grafted linseed oils and their calculated charge densities.

	Grafted Oils					
	MA-10%	MA-20%	MA-30%	MA-DD-24%	MA-DD-42%	MA-DD-55%
Linseed oil % (*w*/*w*)	90.0	80.0	70.0	75.7	58.0	44.6
Maleic anhydride (MA) % (*w*/*w*)	10.0	20.0	30.0	8.4	14.5	19.1
*n*-dodecanol (DD) % (*w*/*w*)	0	0	0	15.9	27.5	36.3
MA + DD % (*w*/*w*)	-	-	-	24.3	42.0	55.4
Charge density ^1^ (mol/kg)	2.0	4.0	6.0	0.8	1.5	1.9

^1^ The potential charge density in mol charges/kg oil, assuming that all the acid groups are dissociated.

**Table 2:** Information about the filaments and their preparation conditions.

	Commercial Alginate						Extracted Alginate			Commercial Alginate						
	Grafted Linseed Oil							Calcium Chloride							Calcium Oxalate	Calcium Phytate
	MA-DD-24%	MA-DD-42%	MA-DD-55%	MA-10%	MA-20%	MA-30%										
Initial alginate conc. (% *w*/*w*)	3	3	3	3	3	3	1.83	2	2	2	3	5	2	5	2	2
Drawing CaCl_2_ conc. (M)	0.02	0.02	0.02	0.02	0.02	0.02	0.02	0.02	0.25	0.02	0.02	0.09	0.09	0.25	0.02	0.02
Final filament diameter (μm)	191	203	2017	202	245	241	175	131	134	163	160	215	182	256	146	156
Excess calcium salt (% *w*/*w*)	33	33	33	33	33	33	33	0	39	0	0	7	22	30	6	9
Sample denotation	A	B	C	D	E	F	G	H	I	J	K	L	M	N	O	P

**Table 3:** Information about the tested filaments and number values for the tensile data. The table also includes calculations of the Δstress-at-break between the adjusted and the calculate stress-at-break, i.e., Equation (7) minus Equation (8). The Δstress-at-break is reported as MPa, MPa per percent of added salt (*) and the percentage change of stress-at-break values per percent of added salt (**).

	Commercial Alginate						Extracted Alginate			Commercial Alginate						
	Grafted Linseed Oil							Calcium Chloride							Calcium Oxalate	Calcium Phytate
	MA-DD-24%	MA-DD-42%	MA-DD-55%	MA-10%	MA-20%	MA-30%										
Excess calcium salt (% *w*/*w*)	33	33	33	33	33	33	33	0	39	0	0	7	22	30	6	9
Dry filament drawing stress with/without excess salt (MPa)	5.27	4.67	4.08	4.71	3.2	3.31	6.28	11.21	10.71	7.24	7.51	4.16	5.81	2.94	9.02	7.90
	7.87	6.97	6.1	7.04	4.78	4.94	9.37	11.21	15.99	7.24	7.51	4.57	7.45	4.38	9.6	8.69
Measure stress-at-break higher average/average/STD (MPa)	246	264	260	229	227	216	262	521	214	447	429	380	298	157	386	317
	240	252	249	223	215	212	251	507	210	441	400	369	274	145	364	310
	5.5	7.7	6.9	5.1	7.1	3.6	6.3	14	4.9	8.6	29.3	7.1	24.7	2.8	14.5	8.3
Calculated stress-at-break with/without excess salt (Equations (6) and (7)) (MPa)	388	367	3434	368	301	306	419	529	513	447	450	368	390	307	468	444
	457	437	414	439	371	377	489	n.s.	600	n.s.	n.s.	381	434	370	479	461
Measured stress-at-break, excess salt adjusted (Equation (8)) (MPa)	368	394	388	342	338	323	391	n.s	350	n.s	n.s.	409	382	224	411	348
Δstress-at-break by the added excess salt (Equation (7) – Equation (8)) (MPa)	91	43	26	96	33	54	99	n.s.	249	n.s.	n.s.	−28	51	146	68	113
Δstress-at-break by the added excess salt (Equation (7) – Equation (8)) (MPa/%) *	2.7	1.3	0.8	2.9	1	1.6	3.9	n.s.	6.4	n.s.	n.s.	−4	2.3	4.9	11.3	12.5
Δstress-at-break by the added excess salt (Equation (7) – Equation (8)) (%/%) **	0.6	0.3	0.2	0.7	0.3	0.4	0.6	n.s.	1.1	n.s.	n.s.	−1	0.5	1.3	2.4	2.7
Moisture absorption after 3 days in 50% air humidity (% *w*/*w*)	8.5	9.9	9.4	8.4	10.6	10.6	n.m.	n.m.	14	n.m.	11.8	n.m.	n.m.	10.3	11.8	12.8
Sample denotation	A	B	C	D	E	F	G	H	I	J	K	L	M	N	O	P

## Data Availability

Not applicable.
